# Relish2 mediates bursicon homodimer-induced prophylactic immunity in the mosquito *Aedes aegypti*

**DOI:** 10.1038/srep43163

**Published:** 2017-02-22

**Authors:** Hongwei Zhang, Shengzhang Dong, Xi Chen, David Stanley, Brenda Beerntsen, Qili Feng, Qisheng Song

**Affiliations:** 1 Division of Plant Sciences, University of Missouri, Columbia, Missouri, USA; 2 USDA/Agricultural Research Service, Biological Control of Insects Research Laboratory, Columbia, Missouri, USA; 3 Department of Veterinary Pathobiology, University of Missouri, Columbia, Missouri, USA; 4 Guangzhou Key Laboratory of Insect Development Regulation and Application Research, School of Life Sciences, South China Normal University, Guangzhou, China

## Abstract

Bursicon is a neuropeptide hormone consisting of two cystine-knot proteins (burs α and burs β), responsible for cuticle tanning and other developmental processes in insects. Recent studies show that each bursicon subunit forms homodimers that induce prophylactic immunity in *Drosophila melanogaster*. Here, we investigated the hypothesis that bursicon homodimers act in prophylactic immunity in insects, and possibly arthropods, generally, using the mosquito, *Aedes aegypti*. We found that *burs α* and *burs β* are expressed in larvae, pupae and newly emerged adults. Treating newly emerged *Ae. aegypti* and *D. melanogaster* adults with recombinant bursicon (r-bursicon) heterodimer led to cuticle tanning in both species. Treating larvae and adults with r-bursicon homodimers led to up-regulation of five anti-microbial peptide (AMP) genes, noting the possibility that bursicon heterodimers also lead to up-regulation of these genes can not been excluded. The induced AMPs effectively suppressed the growth of bacteria *in vitro*. RNAi knock-down of the transcriptional factor Relish2 abolished the influence of r-bursicon homodimers on AMP production. We infer the bursicon homodimers induce expression of AMP genes via Relish2 in *Ae. aegypti*, as prophylactic immunity to protect mosquitoes during the vulnerable stages of each molt.

Insects and other arthropods periodically shed their old cuticles and form new ones during their development to adulthood^1^. The newly-formed cuticle is usually soft and light-colored, vulnerable to injury and pathogen infection. Tanning (hardening and darkening) of new cuticle must occur after each molt in order for insect to survive, and this process is regulated by a neuropeptide hormone bursicon^2^. Bursicon (from the Greek, pertaining to tanning) was first discovered as a bioactive peptide hormone responsible for cuticle tanning immediately after eclosion in blow flies^3^,^4^. About 40 years later, two parallel landmark studies reported that the functional bursicon is a heterodimer consisting of two cysteine-knot protein subunits, named bursicon (burs α) and partner of bursicon (burs β)^5^,^6^. The bursicon heterodimer acts via a specific *Drosophila* leucine-rich G protein-coupled receptor 2 (DLGR2), encoded by the gene *rickets*^7^,^8^. Once activated, DLGR2 stimulates production of cAMP^5^,^6^ and activation of cAMP-dependent protein kinase, resulting in phosphorylation of tyrosine hydroxylase^9^, which in turn regulates the conversion of tyrosine to 3,4-dihydroxyphenylanaline in the metabolic pathways leading to cuticle tanning. Aside from regulating cuticle tanning, bursicon heterodimer acts in several stages of wing expansion and maturation in *Drosophila* and other insect species^10^,^11^,^12^. In *Tribolium,* bursicon signaling is required for integumentary development and adult eclosion, in addition to its roles in cuticle tanning and wing expansion^13^. Bursicon/DLGR2 signaling also acts in migration of the border cells in *Drosophila* during oogenesis^14^.

In addition to forming heterodimers, bursicon subunits form burs α-α and burs β-β homodimers *in vitro*^5^,^15^. Expression profile studies from several insects, including *D. melanogaster, Manduca sexta, Musca domestica* and *Teleogryllus commodus*, report that some neurons exclusively express burs α or burs β^2^,^5^,^11^,^16^. In the blue crab *Callinectes sapidus*, burs β transcripts outnumber burs α transcript by three-fold^17^. These results raised the possibility of bioactive bursicon homodimers, although the biological functions(s) of the homodimers was unknown. Recently, An *et al*.^18^ reported the homodimers mediate innate, prophylactic immunity, registered as induced expression of antimicrobial peptide (AMP) genes during molting in *D. melanogaster.* The homodimers activate a transcriptional factor, Relish, through a DLGR2-independent mechanism^18^. Another recent study reported that burs α (without burs β) influences proliferation of intestinal stem cells via DLGR2 activity in adult *Drosophila* midguts^19^,^20^.

The bursicon homodimer action in prophylactic immunity has not been reported in arthropods other than *D. melanogaster*. Because bursicon is conserved among arthropods, we posed the hypothesis that bursicon homodimers act in prophylactic immunity in insects, and possibly arthropods, generally. We tested our hypothesis in the mosquito, *Aedes aegypti*, a vector of disease organisms, such as yellow fever and Dengue fever. Here, we report on the outcomes of experiments designed to test our hypothesis.

## Results

### AMP expression correlates with bursicon expression during selected life stages of Ae. aegypti

**Figure 1:**
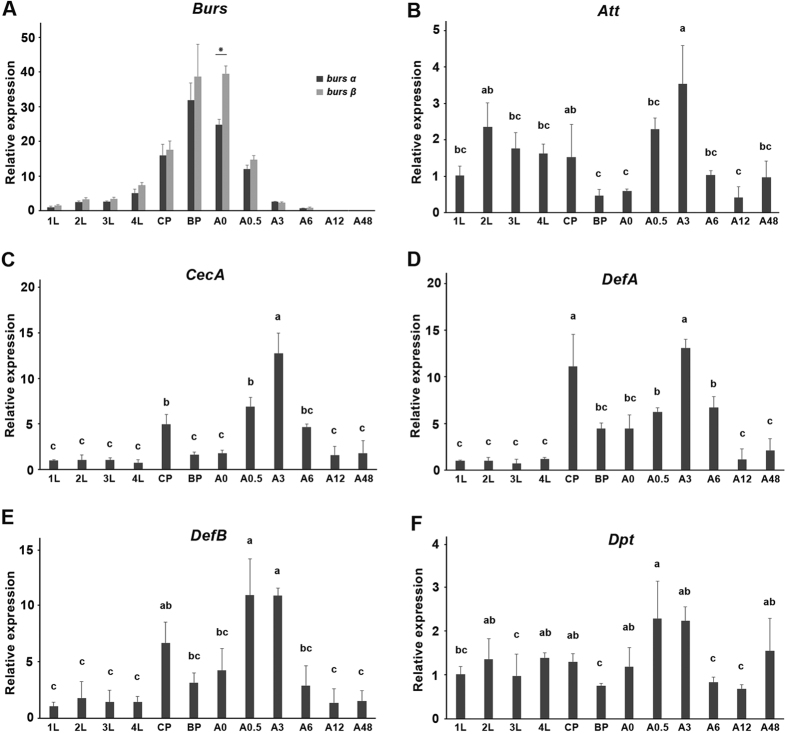
Expression of bursicon transcript (**A**) and AMP genes (**B–F**) during the indicated life stages. *Att, attacin; CecA, Cecropin A; DefA, Defensin A; DefB, Defensin B; Dpt, Diptericin*. The histogram bars represent mean relative accumulation of mRNA encoding each burs subunit, error bars indicate 1 SD, n = 3 independent biological replications. The asterisk indicates significant difference (*P < 0.05). Bars annotated with the same letter are not significantly different, *P* < 0.05. Data are normalized to the expression level of burs α or each AMP gene of 1st instar larvae. 1 L, 1st instar larvae; 2 L, 2nd instar larvae; 3 L, 3rd instar larvae; 4 L, 4th instar larvae; CP, callow pupae; BP, black pupae; A0, adult 0 h; A0.5, adult 0.5 h; A3, adult 3 h; A6, adult 6 h; A12, adult 12 h; A48, adult 48 h.

The accumulation of mRNA encoding both bursicon subunits was determined in larval, pupal and early adult stages. Transcripts of both subunits were negligible in eggs (CTs > 34), then increased through larval and pupal development, and reached a peak level at the black pupal stage. mRNA accumulation rapidly declined to undetectable levels within the first 8 h after adult eclosion (Fig. 1A). The patterns for both subunits were similar, although there were more burs β transcripts at most time points, particularly in newly-emerged adults.

The expression profiles of five AMP genes, including *Attacin (Att*), *Cecropin A (CecA*), *Defensin A (DefA*), *Defensin B (DefB*) and *Dptericin (Dpt*) are shown in Fig. 1B–F. The transcripts of all five AMPs increased at 0.5 h or 3 h after adult eclosion, correlating with the production and release of bursicon after eclosion (Fig. 1B–F). A notable increase of AMP transcripts in callow pupae of mosquitoes was also observed for *CecA, DefA* and *DefB* (Fig. 1C–E).

### Recombinant bursicon (r-bursicon) expression and bioassay

**Figure 2:**
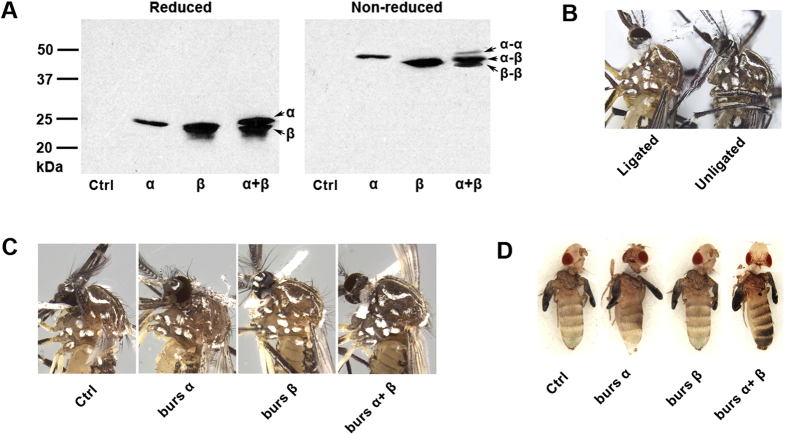
Expression of r-bursicon proteins and bioassay. (**A**) Western blot detection of r-bursicon proteins under reducing (left panel) and non-reducing (right panel) conditions using anti-His-tag antibody. Bursicon monomers formed dimers when expressed *in vitro*. (**B**) Neck-ligation delayed the cuticle tanning in mosquitoes. Photograph was taken at 30 min after emergence. (**C**) r-Bursicon heterodimer induced cuticle tanning in neck-ligated mosquitoes. Photograph was taken at 30 min after r-bursicon treatment. (**D**) *Ae. aegypti* r-busicon treatments led to *D. melanogaster* cuticle tanning. Photograph was taken at 30 min after r-bursicon treatment.

When expressed individually, burs α-α and burs β-β homodimers formed, recognized by their molecular weights, which doubled in the non-reduced gel, compared to the reduced gel (Fig. 2A). Co-expression led to formation of burs α-β heterodimers, as well as small portion of homodimers (Fig. 2A).

Neck-ligating newly-emerged mosquitoes delayed, but did not completely block, cuticle tanning (Fig. 2B). This is probably due to high endogenous phenol oxidase activity. Neck-ligated and untreated mosquitoes completed their tanning by 2–3 h after emergence. The bursicon heterodimer, but not the homodimers, induced cuticle tanning in ligated mosquitoes at 30 min (Fig. 2C), as well as in fruit flies (Fig. 2D), showing the recombinant mosquito bursicon heterodimers were bioactive and functional in the mosquitoes and *D. melanogaster*. We note the cuticle tanning in mosquito is relatively subtle compared to fruit flies, again due to the darker cuticle background in newly-emerged mosquito adults.

### Bursicon homodimers induce AMP gene expression in adult mosquitoes

**Figure 3:**
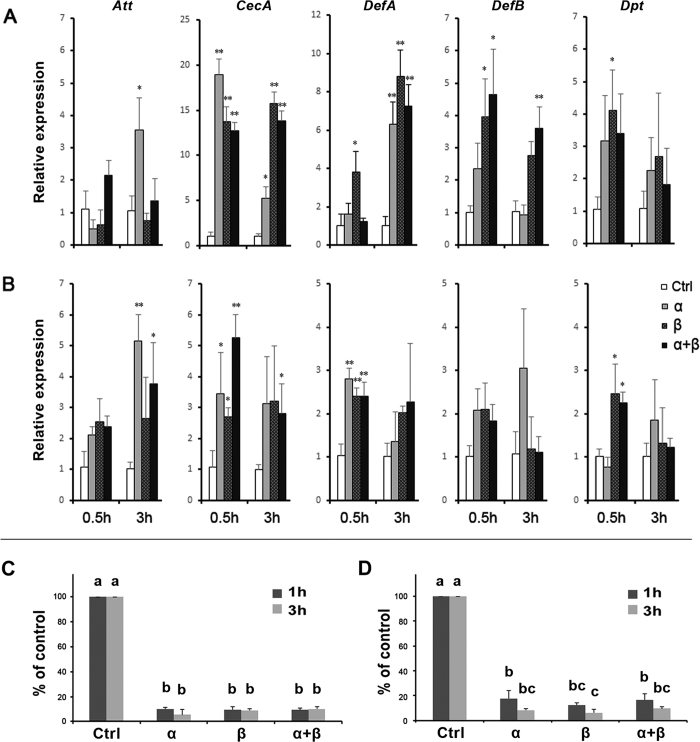
Treatments with r-bursicon homodimers and heterodimer led to up-regulated AMP transcripts in newly-emerged (**A**) and two-day-old mosquitoes (**B**) at 0.5 h or 3 h. *Att, attacin; CecA, Cecropin A; DefA, Defesin A; DefB, Defensin B; Dpt, Diptericin*. Asterisks indicate significant differences (*P < 0.05; **P < 0.01) from the control group. Induced AMPs in newly-emerged mosquitoes significantly suppressed the growth of Gram^-^ bacteria *E. coli* (**C**) or Gram^+^ bacteria *M. luteus* (**D**) *in vitro*. Bars annotated with the same letter are not significantly different, *P* < 0.05.

Compared to controls, separate injections of r-bursicon homodimers and heterodimer induced accumulation of mRNAs encoding the five AMP genes, although the time points and induction intensity differed (Fig. 3A). Among the five AMP genes, mRNAs encoding *CecA* and *DefA* reached the highest levels. Similar results were also found in male mosquitoes (data not shown). The action of bursicon homodimers and heterodimer was also tested in two-day old adults, in which mRNAs encoding the bursicon subunits were depleted. Injected r-bursicon homodimers and heterodimer induced the expression of AMP genes, compared to control group, albeit at lower levels compared to newly-emerged adults (Fig. 3B).

Bacterial inhibition assays using newly-emerged adult preparations showed that the growth of *Escherichia coli* was significantly inhibited in samples treated with r-bursicon homodimers and heterodimer, with 5–10% surviving bacteria, compared to the control group (Fig. 3C). Growth of *Micrococcus luteus* was similarly inhibited, noting the inhibitory effect was reduced compared to *E. coli*, with 6–18% surviving bacteria, compared to the control group (Fig. 3D).

### Bursicon homodimers induce AMP expression in larval fat body (FB)

**Figure 4:**
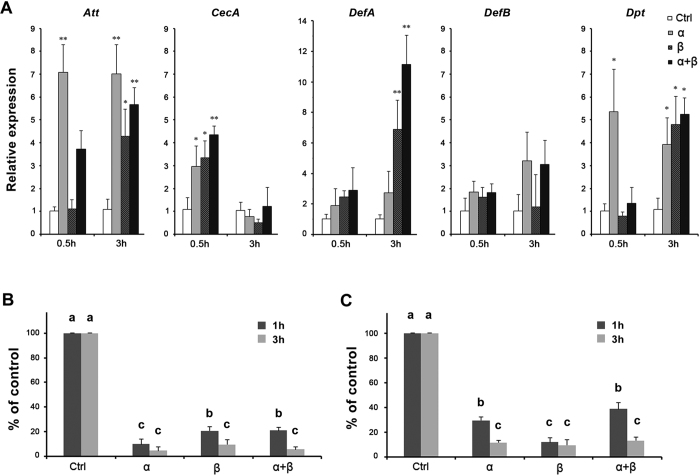
Treatments with r-bursicon homodimers and heterodimer induced AMP production in isolated larval fat body preparations. (**A**) Accumulation of mRNA encoding five AMP genes following treatments with r-bursicon homodimers or heterodimer. Asterisks indicate significant differences (*P < 0.05; **P < 0.01) from the control group. The growth of *E. coli* (**B**) and *M. luteus* (**C**) was significantly suppressed by induced larval fat body AMPs. Bars annotated with the same letter are not significantly different, *P* < 0.05.

After incubation with r-bursicon homodimers or r-heterodimer, transcripts encoding the five AMP genes just mentioned were up-regulated in larval FB preparations by 2–10 fold (Fig. 4A), congruent with the results from adult mosquitoes. The bacterial inhibition assay using larval FB preparations significantly suppressed the growth of *E. coli* (Fig. 4B) and *M. luteus* (Fig. 4C).

### Bursicon homodimers induce AMP expression via Relish2

**Figure 5:**
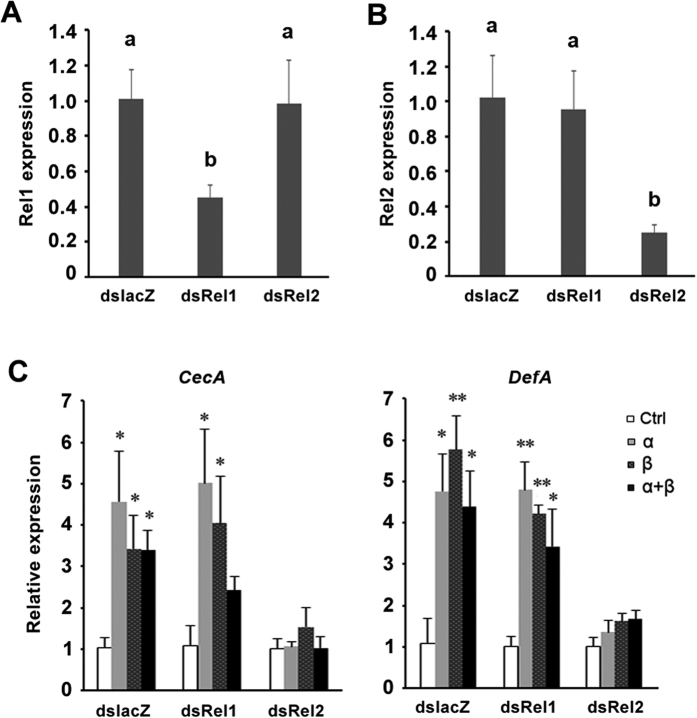
RNAi knockdown of *Relish 2* led to reduced expression of AMP genes induced by r-bursicon homodimers or heterodimer. (**A**) dsRelish 1 treatments led to gene-specific reduction of mRNA encoding *Relish 1*, but not *Relish 2*. (**B**) dsRelish 2 treatment led to gene-specific reduction of mRNA encoding *Relish 2*, but not *Relish 1*. Bars annotated with the same letter are not significantly different, *P* < 0.05. (**C**) The induction of *CecA* and *DefA* by r-bursicon homodimers and heterodimer was attenuated by dsRelish2, but not dsRelish 1. Asterisks indicate significant differences (*P < 0.05; **P < 0.01) from the control group.

Newly-emerged female mosquitoes were injected with ds*Rel1* or ds*Rel2*, and the relative accumulation of their transcripts was quantified at 48 h PT. *Rel1* and *Rel2* transcripts were reduced by 62% and 75% respectively, compared to controls (Fig. 5A,B). r-Bursicon homodimers and heterodimer were injected into dsRNA-treated mosquitoes, and relative accumulation of *CecA* and *DefA* transcripts were determined at 0.5 h. In the ds*lacZ* treated mosquitoes (control), r-bursicon induced gene expression of *CecA* and *DefA*, similar to the results in two-day old mosquitoes (Fig. 5C). *Rel1* knock-down did not influence the induction of *CecA* and *DefA*, compared to control group (*P* > 0.05). However, *Rel2* knock-down totally abolished the up-regulation of *CecA* and *DefA* induced by r-bursicon homodimers or heterodimer (*P* < 0.001).

## Discussion

The data reported in this paper support our hypothesis that burs homodimers act in prophylactic immunity of insects, and possibly arthropods, generally. Several points lead to this sound conclusion. First, the relative expression of each burs subunit increased from very low in first instar larvae to substantially high in pupae, then rapidly declined after adult emergence. The increase in AMP gene expression after adult eclosion correlates with bursicon release. Second, r-bursicon formed r-burs α-α and r-burs β-β homodimers in an *in vitro* expression system. The r-burs α-β heterodimer, but not homodimers, was biologically active, leading to cuticle tanning in newly-emerged *Ae. aegy*pti and *D. melanogaster* adults. Third, treating newly emerged and day two adult mosquitoes with the r-bursicon homodimers and heterodimer led to rapid and sustained induction of genes encoding AMPs. Fourth, the induced AMPs were biologically active, leading to inhibition of Gram-negative and Gram-positive bacterial growth. Finally, the r-bursicon homodimers and -heterodimer induced expression of genes encoding AMPs via Relish 2. Taken together, these points support our inference that bursicon homodimers mediate expression of AMPs in newly emerged adult mosquitoes, as reported for *D. melanogaster*^18^.

Insect innate immunity is a generalized reaction to wounds, infections and invasions that does not depend on previous immune experiences. In broad terms, it is composed of a surveillance system that detects a challenge and initiates immune signaling pathways, such as Toll and IMD, responsible for launching cellular and humoral responses to challenge^21^. Here, we emphasize the bursicon homodimer-induced up-regulation of genes encoding AMPs is not a response to immune challenge, but hormone-regulated prophylactic immunity expressed during times of extraordinary vulnerability, when newly-formed cuticle is soft and easily penetrated or wounded. Another instance of prophylactic immunity was recorded in tobacco hornworm, *Manduca sexta*, pupae. Low levels of lysozyme were present in midgut cells of early fifth-instar larvae, which increased to high levels later in the instar, accumulated in large vacuoles in regenerative cells of the midgut. These cells form a continuous layer underneath the larval midgut cells. At the pupal molt the larval midgut is released and the regenerative cells remain as a single layer of cells, which release the high levels of lysozyme into the lumen of the pupal midgut. The authors inferred the high lysozyme activity in the pupal midgut results from developmental regulation of the midgut lysozyme gene to provide prophylactic protection from specific bacterial infections^22^. In a similar vein, Moret and Siva-Jothy reported that mealworm beetles, *Tenebrio molitor*, express immune responses that persist for long enough to provide prophylaxis from secondary infections^23^. This is adaptive because it provides a survival benefit to the larvae. We speculate that various forms of prophylactic immunity await discovery among invertebrates.

Bursicon consists of two subunits, burs α and burs β, members of the cystine-knot protein (CKP) family^5^. One common feature of CKPs is that they usually form dimers, either homo- or heterodimers, responsible for certain biological function, by binding to specific receptors^24^. The bursicon heterodimer acts through DLGR2 to mediate various biological processes, including cuticle tanning, wing expansion, integumentary development and boarder cell migration^2^,^13^,^14^,^25^. To date, the bursicon heterodimer, but not bursicon homodimers, activates DLGR2 and initiates the insect cuticle tanning. Recently, An and coworkers reported that both bursicon homodimers are involved in regulating immune responses, by inducing a set of AMPs and stress-related genes, in a DLGR2-independent manner^18^. Involvement of cystine-knot protein family members in insect innate immunity has been demonstrated in *Drosophila*. For example, the role of spätzle, the cystine-knot protein homodimer (active form), in regulating the immune response in the Toll signaling pathway in *Drosophila*, is established^26^. Very recently, it is reported that nerve growth factor β (NGF β), another cystine-knot protein superfamily member, regulates the immune response in vertebrates^27^.

The amino acid sequences of both bursicon subunits are highly conserved among arthropods and echinoderms^2^,^28^. We speculate the function of bursicon/DLGR2 signaling is conserved among these animals, to generate hard, protective outer cuticles, among a growing list of actions. Because the amino acid sequences are highly conserved, it is reasonable that the *Ae. aegypti* bursicon can initiate cuticle tanning in *Drosophila*. Bursicon from diverse insects and the lobster, *Homarus americanus*, also induce tanning in *Drosophila*^2^,^29^,^30^. Lepidopteran bursicon does not induce tanning in *D. melanogaster*, probably due to low amino acid sequence identity^11^. The function of bursicon homodimer signaling in immunity is probably conserved among insects, at least in Dipterans, because bursicon homodimers induce AMP production through Relish2 in mosquitoes, as seen in *D. melanogaster*.

As mentioned, expression of transcripts encoding both bursicon subunits occurred in similar patterns in larvae, pupae and adults. We infer that heterodimers and homodimers are formed as observed *in vitro*. A similar expression pattern has been reported in another mosquito, *An. gambiae*^31^. Although the expression patterns of bursicon subunits are similar, there are differences, which we take to indicate precise control of subunit expression. Burs β transcripts outnumber burs α ones, and this is similar to results obtained from the blue crab, *C. sapidus*, where burs β transcripts outnumber burs α transcripts^17^. We infer the large abundance of burs β transcripts promote formation of the burs β-β homodimer. Here, we infer the increased transcripts of all five AMPs after adult eclosion indicate AMP production is directly linked to bursicon release after each molt.

We used r-bursicon homodimers and heterodimer to investigate their actions in mosquitoes. Treatments with bursicon homodimers or heterodimer led to a general pattern of up-regulating the five AMP genes in mosquito larvae, newly-emerged adults and two-day old adults. We take these five AMP genes as a sub-set of the 15 bursicon homodimer-induced AMP genes in our *D. melanogaster* study^18^, from which we propose that bursicon homodimers induce more than five AMP-encoding genes in *Ae. aegypti*. It is interesting that a strong induction of AMP genes is observed shortly after 0.5 h treatment of r-bursicon homodimers or heterodimer, and this quick transcriptional response suggests bursicon homodimers signaling can rapidly lead to relish activation. The bacteria inhibition assay confirmed the induction of AMPs at protein level, effectively preventing bacterial infection by inducing AMP production. The induction of AMPs in two-day old adults suggests the bursicon-mediated AMP up-regulation continues into adulthood, although it is not yet clear how long into adulthood. It may not continue far, however, because the induction of AMP expression is reduced in two-day adults, compared to larvae and newly-emerged adults.

Our results showed both bursicon homodimers and heterodimer induced AMP production in *Ae. aegypti*; however, it is most likely the induction of AMP expression is attributed to the bursicon homodimers. We make this speculation for two reasons: first, appreciable amount of bursicon homodimers was presented in the bursicon heterodimer sample; second, the absence of DLGR2 signaling in a DLGR2 mutant *D. melanogaster* did not influence the immune-inducing role of bursicon^18^. Nevertheless, bursicon signaling in *Ae. aegypti* may differ from *D. melanogaster*, and the role of bursicon heterodimer in inducing AMP production is not excluded.

We investigated the role of a transcriptional factor, Relish, responsible for the expression of many AMPs. Unlike *D. melanogaster, Ae. aegypti* has two Relishes, Relish1 and Relish2, homologues of *D. melanogaster* Dorsal and Relish, respectively^32^,^33^,^34^. Our results indicate that Relish2, but not Relish1, acts in bursicon homodimer-induced AMP expression. This also suggests in *Ae. aegypti* that the IMD, but not Toll, pathway is activated by bursicon homodimers as reported in *Drosophila*^18^. Recent studies^33^,^35^,^36^ on mosquito Relish have shown that Relish1 is involved in the response to fungal infections, while Relish2 participates in protection against Gram-positive and Gram-negative bacteria.

The findings in this report show that the biological significance of bursicon extends beyond its classical developmental roles in cuticle tanning and wing expansion to mediating prophylactic immunity during molting periods. Taken with other recent reports of bursicon activities, we foresee future discovery of a fairly wide range of bursicon-mediated events in insects and other arthropods. Identification of the important homodimer receptor is currently underway.

## Materials and Methods

### Mosquito and fruit fly maintenance

*Ae. aegypti* Liverpool (LVP) strain used in this study was maintained as described^37^. Briefly, mosquito larvae were reared in plastic pans with TetraMin diet under our standard conditions at 26 °C; adult mosquitoes were raised in an environmental chamber at 80 ± 5% humidity, 26 ± 2 °C and 16L:8D photoperiod.

*Drosophila melanogaster* Oregon-R strain used in this study was maintained on blue culture medium (Fisher Scientific) at 20 °C.

### RNA extraction and cDNA synthesis

Total RNA was isolated from larval FB preparations or whole larval, pupal and adult mosquitos using TRIzol reagent (Invitrogen), according to the manufacturer’s protocol. Two μg RNA was used to synthesize the first strand cDNA using the GoScript™ Reverse Transcription Kit (Promega), following the manufacturer’s instructions.

### r-Bursicon protein expression and purification

Specific primers with restriction sites (Supplementary Table S1) were designed to generate DNA fragments encoding the *Ae. aegypti* bursicon proteins. The fragments were subcloned into pcDNA3.1 expression plasmid and confirmed by DNA sequencing. Mammalian HEK293T cells were obtained from Dr. Christian Lorson (University of Missouri) and maintained in complete Dulbecco’s modified Eagle medium (DMEM with 10% FBS) in a 5% CO_2_ incubator at 37 °C. The constructed pcDNA3.1 plasmids were transfected into mammalian HEK293T cells either individually or simultaneously using the SatisFection™ Transfection Reagent (Agilent Technologies). Control cells were transfected with a pcDNA3.1 blank vector. After 24 h, the complete DMEM was replaced with serum-free DMEM. At 48–72 h after transfection, the culture medium was collected and centrifuged at 3,000× g to remove the cell debris. Ni-NTA His-bind resin (QIAGEN) was used to enrich the r-bursicon proteins from the supernatant, and identities of the proteins were verified by Western blot using a His-tag antibody. Culture media for the control cells were similarly prepared for bioassays, described just below.

### Bursicon homodimer or heterodimer treatment and bioassay

Mosquitoes and fruit flies were neck-ligated immediately after eclosion, and incubated 30 min to ensure complete ligation. The r-bursicon homodimers or heterodimer were injected into the thorax of the flies (0.5 μL; 40 ng/μL), using a microinjection system equipped with a hand-calibrated pulled glass needle. Control mosquitoes were injected with media prepared from the blank-vector transfected cells, which had been purified using the same procedure. Two-day old adult mosquitoes without ligation were similarly injected with the r-bursicon homodimers or heterodimer. At the indicated times post treatment (PT), the state of cuticle tanning was recorded and RNA extracted for qRT-PCR.

### Larval fat body incubation

Larval (4^th^ instar) mosquito FBs were isolated in Ringer’s solution, and washed twice with Ringer’s solution. FBs (10 per group) were incubated in 150 μL Grace’s insect medium (Lonza, USA) containing 80 ng of r-bursison (rburs α, rburs β or rburs α + β) or control media preparations for 0.5, 1 or 3 h. After incubation, FB preparations were processed for RNA extraction as described or for bacterial inhibition assays, as described below.

### Bacterial inhibition assay

The bacterial inhibition assay was performed as previously described with slight modification^18^. Larval FB samples were homogenized on ice and centrifuged at 16000× g for 20 min at 4 °C, at 1 h or 3 h after incubation with r-bursicon homodimers or heterodimer as described above. Newly-emerged, bursicon-treated mosquitoes were homogenized at 1 h or 3 h after injection in Grace’s insect medium, and then centrifuged at 16000× g for 20 min at 4 °C. The resulting supernatants from ten larval FB body or newly-emerged adults were mixed with 1 × 10^4^ Gram-negative bacteria *E. coli* or Gram-positive bacteria *M. luteus*. After incubation at 37 °C for 3 h, the mixtures were plated onto LB agar plates and the bacterial colonies were counted after overnight incubation at 37 °C.

### Quantitative real-time polymerase chain reaction (qRT-PCR)

Specific primers (Supplementary Table S1) were designed to detect the bursicon genes (burs α and burs β) or antimicrobial peptide (AMP) genes, including *Att, CecA, DefA, DefB* and *Dpt*. The qPCR amplification was carried out on an Applied Biosystems (ABI) 7500 Fast Real-Time PCR System following the protocol of ABI SYBR green Supermix (ABI), programmed at 95 °C for 3 min, followed by 40 cycles of 95 °C for 10 s and 68 °C for 30 s. The melt curves from 60 °C to 95 °C were determined. The RPL8 gene was used as a reference gene for an internal RNA control^37^,^38^, n = 3 biologically independent replicates. Relative mRNA accumulation was determined by the 2^−ΔΔCT^ method (ΔΔCT = ΔCT_Target gene_ − ΔCT_RP49_)^39^.

### RNA interference

Two pairs of primers (Rel1iF and Rel1iR; Rel2if and Rel2iR^35^, Supplementary Table S1), each linked to the T7 promoter region, were designed to amplify the DNA templates for dsRel1 and dsRel2 synthesis. dsRNA was synthesized using the MEGAscript Kit (Ambion), according to the manufacturer’s instructions. DNase I was used to remove the templates, and synthesized dsRNA was then purified by phenol/chloroform extraction and ethanol precipitation. The purified product was resuspended in ddH_2_O and checked on agarose gel to ensure the purity of dsRNA. To exclude the nonspecific effect of dsRNA injection, dsRNA synthesized using the bacterial β-galactosidase (lacZ) gene DNA was employed as a control^35^. The procedures for synthesis and purification of dslacZ RNA were the same as described above.

Mosquitoes used for dsRNA injection were newly-emerged female adults. The injection of dsRNAs (500 ng/mosquito) was performed similar to the bursicon injection described above. Injected mosquitoes were kept in our standard conditions. At 48 h PT, mosquitoes were treated with r-burs (burs α, burs β or burs α + β) or control proteins. At 0.5 h after treatment, the mosquitoes were processed for RNA extraction.

### Statistical analysish

For our transcriptional analyses of AMP expression following bursicon treatment, data obtained from qRT-PCR were analyzed using the Student’s paired *t*-test, and *P* < 0.05 was considered statistically significant. For AMP expression at different life stages and bacterial inhibition assay, One-way ANOVA, followed by Tukey’s test (*P* < 0.05), was used to evaluate significant differences among different treatment groups. For comparison between AMP expression between different dsRNA-treatment groups, we use two-way ANOVA and Turkey’s *post hoc* test (*P* < 0.05).

## Additional Information

**How to cite this article:** Zhang, H. *et al*. Relish2 mediates bursicon homodimer-induced prophylactic immunity in the mosquito *Aedes aegypti. Sci. Rep.* **7**, 43163; doi: 10.1038/srep43163 (2017).

**Publisher's note:** Springer Nature remains neutral with regard to jurisdictional claims in published maps and institutional affiliations.

## Supplementary Material

Supplementary Table S1

## References

[b1] Hesterlee S. & Morton D. B. Insect physiology: the emerging story of ecdysis. Curr Biol 6, 648–650 (1996). 8793284 10.1016/s0960-9822(09)00439-4

[b2] Honegger H. W., Dewey E. M. & Ewer J. Bursicon, the tanning hormone of insects: recent advances following the discovery of its molecular identity. J Comp Physiol A Neuroethol Sens Neural Behav Physiol 194, 989–1005 (2008). 19005656 10.1007/s00359-008-0386-3

[b3] Fraenkel G. H. C. Hormonal and nervous control of tanning in the fly. Science 138, 27–29 (1962). 13894424 10.1126/science.138.3536.27

[b4] Cottrell C. B. The imaginal ecdysis of blowflies. The control of cuticular hardening and darkening. J Exp Biol 39, 395–411 (1962).

[b5] Luo C. W. . Bursicon, the insect cuticle-hardening hormone, is a heterodimeric cystine knot protein that activates G protein-coupled receptor LGR2. Proc Natl Acad Sci USA 102, 2820–2825 (2005). 15703293 10.1073/pnas.0409916102 PMC549504

[b6] Mendive F. M. . *Drosophila* molting neurohormone bursicon is a heterodimer and the natural agonist of the orphan receptor DLGR2. FEBS Lett 579, 2171–2176 (2005). 15811337 10.1016/j.febslet.2005.03.006

[b7] Baker J. D. & Truman J. W. Mutations in the *Drosophila* glycoprotein hormone receptor, rickets, eliminate neuropeptide-induced tanning and selectively block a stereotyped behavioral program. J Exp Biol 205, 2555–2565 (2002). 12151362 10.1242/jeb.205.17.2555

[b8] Dewey E. M. . Identification of the gene encoding bursicon, an insect neuropeptide responsible for cuticle sclerotization and wing spreading. Curr Biol 14, 1208–1213 (2004). 15242619 10.1016/j.cub.2004.06.051

[b9] Davis M. M., O’Keefe S. L., Primrose D. A. & Hodgetts R. B. A neuropeptide hormone cascade controls the precise onset of post-eclosion cuticular tanning in Drosophila melanogaster. Development 134, 4395–4404 (2007). 18003740 10.1242/dev.009902

[b10] Natzle J. E., Kiger J. A. Jr. & Green M. M. Bursicon signaling mutations separate the epithelial-mesenchymal transition from programmed cell death during *Drosophila melanogaster* wing maturation. Genetics 180, 885–893 (2008). 18780731 10.1534/genetics.108.092908 PMC2567388

[b11] Dai L. . Identification, developmental expression, and functions of bursicon in the tobacco hawkmoth, Manduca sexta. J Comp Neurol 506, 759–774 (2008). 18076057 10.1002/cne.21575

[b12] Huang J. . RNA interference-mediated silencing of the bursicon gene induces defects in wing expansion of silkworm. FEBS Lett 581, 697–701 (2007). 17270178 10.1016/j.febslet.2007.01.034

[b13] Bai H. & Palli S. R. Functional characterization of bursicon receptor and genome-wide analysis for identification of genes affected by bursicon receptor RNAi. Dev Biol 344, 248–258 (2010). 20457145 10.1016/j.ydbio.2010.05.003 PMC2909337

[b14] Anllo L. & Schupbach T. Signaling through the G-protein-coupled receptor Rickets is important for polarity, detachment, and migration of the border cells in *Drosophila*. Dev Biol 414, 193–206 (2016). 27130192 10.1016/j.ydbio.2016.04.017 PMC4887387

[b15] An S. . Global identification of bursicon-regulated genes in *Drosophila melanogaster*. BMC Genomics 9, 424 (2008). 18801173 10.1186/1471-2164-9-424 PMC2566319

[b16] Wang S., An S. & Song Q. Transcriptional expression of bursicon and novel bursicon-regulated genes in the house fly *Musca domestica*. Arch Insect Biochem Physiol 68, 100–112 (2008). 18454490 10.1002/arch.20239

[b17] Chung J. S., Katayama H. & Dircksen H. New functions of arthropod bursicon: inducing deposition and thickening of new cuticle and hemocyte granulation in the blue crab, *Callinectes sapidus*. PLoS One 7, e46299 (2012). 23029467 10.1371/journal.pone.0046299 PMC3460823

[b18] An S. . Insect neuropeptide bursicon homodimers induce innate immune and stress genes during molting by activating the NF-kappaB transcription factor Relish. PLoS One 7, e34510 (2012). 22470576 10.1371/journal.pone.0034510 PMC3314635

[b19] Scopelliti A., Bauer C., Cordero J. B. & Vidal M. Bursicon-alpha subunit modulates dLGR2 activity in the adult *Drosophila melanogaster* midgut independently to Bursicon-beta. Cell Cycle 15, 1538–1544 (2016). 27191973 10.1080/15384101.2015.1121334 PMC4934083

[b20] Scopelliti A. . Local control of intestinal stem cell homeostasis by enteroendocrine cells in the adult *Drosophila* midgut. Curr Biol 24, 1199–1211 (2014). 24814146 10.1016/j.cub.2014.04.007 PMC4046228

[b21] Lemaitre B. & Hoffmann J. The host defense of *Drosophila melanogaster*. Annu Rev Immunol 25, 697–743 (2007). 17201680 10.1146/annurev.immunol.25.022106.141615

[b22] Russell V. W. & Dunn P. E. Lysozyme in the midgut of *Manduca sexta* during metamorphosis. Arch Insect Biochem Physiol 17, 67–80 (1991). 1802032 10.1002/arch.940170202

[b23] Moret Y. & Siva-Jothy M. T. Adaptive innate immunity? Responsive-mode prophylaxis in the mealworm beetle, *Tenebrio molitor*. Proc Biol Sci 270, 2475–2480 (2003). 14667338 10.1098/rspb.2003.2511 PMC1691523

[b24] Iyer S. & Acharya K. R. Tying the knot: the cystine signature and molecular-recognition processes of the vascular endothelial growth factor family of angiogenic cytokines. FEBS J 278, 4304–4322 (2011). 21917115 10.1111/j.1742-4658.2011.08350.x PMC3328748

[b25] Dong S. . The neuropeptide bursicon acts in cuticle metabolism. Arch Insect Biochem Physiol 89, 87–97 (2015). 25821138 10.1002/arch.21227

[b26] Hoffmann A. . Biophysical characterization of refolded *Drosophila* Spatzle, a cystine knot protein, reveals distinct properties of three isoforms. J Biol Chem 283, 32598–32609 (2008). 18790733 10.1074/jbc.M801815200

[b27] Hepburn L. . Innate immunity. A Spaetzle-like role for nerve growth factor beta in vertebrate immunity to *Staphylococcus aureus*. Science 346, 641–646 (2014). 25359976 10.1126/science.1258705 PMC4255479

[b28] Van Loy T. . Evolutionary conservation of bursicon in the animal kingdom. Gen Comp Endocrinol 153, 59–63 (2007). 17275819 10.1016/j.ygcen.2006.12.004

[b29] Honegger H. W., Dewey E. M. & Kostron B. From bioassays to *Drosophila* genetics: strategies for characterizing an essential insect neurohormone, bursicon. Acta Biol Hung 55, 91–102 (2004). 15270222 10.1556/ABiol.55.2004.1-4.11

[b30] Kostron B., Marquardt K., Kaltenhauser U. & Honegger H. W. Bursicon, the cuticle sclerotizing hormone-comparison of its molecular mass in different insects. J. Insect Physiol. 41, 1045–1053 (1995).

[b31] Honegger H. W., Estevez-Lao T. Y. & Hillyer J. F. Bursicon-expressing neurons undergo apoptosis after adult ecdysis in the mosquito *Anopheles gambiae*. J Insect Physiol 57, 1017–1022 (2011). 21554887 10.1016/j.jinsphys.2011.04.019

[b32] Shin S. W., Kokoza V., Ahmed A. & Raikhel A. S. Characterization of three alternatively spliced isoforms of the Rel/NF-kappa B transcription factor Relish from the mosquito *Aedes aegypti*. Proc Natl Acad Sci USA 99, 9978–9983 (2002). 12119421 10.1073/pnas.162345999 PMC126610

[b33] Shin S. W. . REL1, a homologue of Drosophila dorsal, regulates toll antifungal immune pathway in the female mosquito *Aedes aegypti*. J Biol Chem 280, 16499–16507 (2005). 15722339 10.1074/jbc.M500711200

[b34] Antonova Y., Alvarez K. S., Kim Y. J., Kokoza V. & Raikhel A. S. The role of NF-kappaB factor REL2 in the *Aedes aegypti* immune response. Insect Biochem Mol Biol 39, 303–314 (2009). 19552893 10.1016/j.ibmb.2009.01.007 PMC2702699

[b35] Magalhaes T., Leandro D. C. & Ayres C. F. Knock-down of REL2, but not defensin A, augments *Aedes aegypti* susceptibility to *Bacillus subtilis* and *Escherichia coli*. Acta Trop 113, 167–173 (2010). 19879852 10.1016/j.actatropica.2009.10.013

[b36] Shin S. W., Kokoza V., Lobkov I. & Raikhel A. S. Relish-mediated immune deficiency in the transgenic mosquito *Aedes aegypti*. Proc Natl Acad Sci USA 100, 2616–2621 (2003). 12594340 10.1073/pnas.0537347100 PMC151389

[b37] Wang S. & Beerntsen B. T. Functional implications of the peptidoglycan recognition proteins in the immunity of the yellow fever mosquito, *Aedes aegypti*. Insect Mol Biol 24, 293–310 (2015). 25588548 10.1111/imb.12159

[b38] Lan Q. & Fallon A. M. Sequence analysis of a mosquito ribosomal protein rpL8 gene and its upstream regulatory region. Insect Mol Biol 1, 71–80 (1992). 1343779 10.1111/j.1365-2583.1993.tb00107.x

[b39] Livak K. J. & Schmittgen T. D. Analysis of relative gene expression data using real-time quantitative PCR and the 2 (-Delta Delta C(T)) Method. Methods 25, 402–408 (2001). 11846609 10.1006/meth.2001.1262

