# Supplementary material for: Relish2 mediates bursicon homodimer-induced prophylactic immunity in the mosquito Aedes aegypti
Source: Sci Rep. 2017 Feb 22;7:43163. doi: 10.1038/srep43163 (PMC5320557; doi:10.1038/srep43163)
Supplement: Supplementary Table S1 [file srep43163-s1.pdf]

Relish2 mediates bursicon homodimer-induced prophylactic immunity in the mosquito *Aedes aegypti*

Hongwei Zhang[1], Shengzhang Dong[1], Xi Chen[1], David Stanley[2], Brenda Beerntsen[3], Qili Feng[4], Qisheng Song[1]*

[1] Division of Plant Sciences, University of Missouri, Columbia, Missouri, USA

[2] USDA/Agricultural Research Service, Biological Control of Insects Research Laboratory, Columbia, Missouri, USA

[3] Department of Veterinary Pathobiology, University of Missouri, Columbia, Missouri, USA

[4]Guangzhou Key Laboratory of Insect Development Regulation and Application Research, School of Life Sciences, South China Normal University, Guangzhou, China

*Corresponding author
Dr. Qisheng Song
Division of Plant Sciences
University of Missouri
Columbia, MO 65211
Tel: (573) 882-9798
Email: songq@missouri.edu

Table S1. Sequences of the primers used in this study.

Category	Primer	Direction	Sequence (5′–3′)*
Recombinant bursicon expression	αExF	Forward	TACTCACTCGAGATGAAGTCATCCGTGTGC
	αExR	Reverse	TACTCAGGATCCACCTCCGCAGCTTTGAACTG
	βExF	Forward	TACTCACTCGAGATGTACAGGCTAGAAACA
	βExR	Reverse	TACTCAGGATCCACGCGAACCAGATCGCCACA
Bursicon transcripts detection	αRTF	Forward	AGGATTGAACGCCCAGAAAG
	αRTR	Reverse	GCCCGAGACCTGAATGTAGC
	βRTF	Forward	TAACAGTGAACAAGTGCGAAGG
	βRTR	Reverse	AGGAACGACTCCCGACAGC
AMP transcripts detection	AttF	Forward	GCTGTTCACTTTCGTCTTTCTG
	AttR	Reverse	TTGTTCGTTGACTGACTACCG
	CecAF	Forward	ATTTCTCCTGATCGCCGTGGCTG
	CecAR	Reverse	GAGCCTTCTCGGCGGCATTGAA
	DefAF	Forward	GCCACCTGTGATCTGCTGAGCGGA
	DefAR	Reverse	GGAGTTGCAGTAGCCTCCCCGAT
	DefBF	Forward	TGCCTACCCACAGGAACCG
	DefBR	Reverse	GAGCAGCACAAGCACTATCACC
	DiptAF	Forward	ATCCGATTCAGAATTCGCTTT
	DiptAR	Reverse	TTTACCGTCTCCCTGAAATCC
RNAi	Rel1iF	Forward	*TAATACGACTCACTATAGGGAG*AATCTGGTCGGCAAGGAG
	Rel1iR	Reverse	*TAATACGACTCACTATAGGGAG*GCACGTTGGTATGCTGGAA
	Rel2iF	Forward	*TAATACGACTCACTATAGGGAG*ACCGGTGGAAGTGCTC
	Rel2iR	Reverse	*TAATACGACTCACTATAGGGAG*CCCCGATCTCCGTTAT
	lacZiF	Forward	*TAATACGACTCACTATAGGGAG*ACCCTGGCGTTACCC
	lacZiR	Reverse	*TAATACGACTCACTATAGGGAG*CCCGTTGCACCACAGA
Relish transcripts detection	Rel1RTF	Forward	GCGAGTTCCAGCATACCA
	Rel1RTR	Reverse	CTGAGTCAAGGGGCGTCA
	Rel2RTF	Forward	GGACGAGGCAGCGGCGCAGTTTGAGC
	Rel2RTR	Reverse	TCCAGAGGGCCGAGATAAGTTCC
Reference gene	RPL8F	Forward	ATACCAAGCGTACCCGTGTC
	RPL8R	Reverse	ATGCTCAACGGGGTTCATAG

* The underlined nucleotides indicate the restriction recognition sites. T7 region for RNA polymerase binding is in italics.
